# Congenital Isolated Bilateral Anophthalmia: A Case Report

**DOI:** 10.7759/cureus.111374

**Published:** 2026-06-23

**Authors:** Ikram El Hachmi, Anass Ayyad, Manal Elidrissi Errahhali, Sahar Messaoudi, Rim Amrani

**Affiliations:** 1 Department of Neonatology and Neonatal Resuscitation, Maternal, Child and Mental Health Research Laboratory, Faculty of Medicine and Pharmacy of Oujda, Mohammed First University, Oujda, MAR; 2 Genetics Unit, Medical Sciences Research Laboratory, Faculty of Medicine and Pharmacy, Mohammed First University, Oujda, MAR

**Keywords:** bilateral anophthalmia, congenital anophthalmia, congenital eye anomaly, neonate, ocular malformations, orbital mri

## Abstract

Congenital anophthalmia is the complete absence of the eye resulting from a failure of optic vesicle formation during early embryogenesis. It may present as a unilateral or bilateral condition and can occur as an isolated anomaly or as part of a syndromic disorder. We report the case of a full-term newborn admitted on the second day of life for the management of bilateral congenital anophthalmia. Family history was notable for anophthalmia in three cousins belonging to the shared parental lineage, suggesting a possible genetic predisposition. The diagnosis is primarily clinical and is confirmed by ocular ultrasonography and cranio-orbital magnetic resonance imaging (MRI). Several etiologies may be involved, including genetic mutations, chromosomal abnormalities, intrauterine infections, and exposure to teratogenic agents during pregnancy. The birth of a child with congenital anophthalmia constitutes a significant challenge for both the family and healthcare providers. Optimal management therefore requires a multidisciplinary approach involving ophthalmologists, pediatricians, geneticists, psychologists, and the child's caregivers.

## Introduction

Congenital anophthalmia is defined as the complete absence of the ocular globe due to a failure of optic vesicle formation during early embryonic development, whereas microphthalmia refers to the presence of a hypoplastic or underdeveloped eye at birth. They are associated with variable but often profound visual impairment and may also disrupt normal development of the orbit and facial structures [1]. Together, they represent a rare spectrum of congenital ocular malformations [2,3]. Estimates of birth prevalence vary according to diagnostic definitions and imaging practices, ranging from 0.2-0.4 per 10,000 births for anophthalmia and 0.2-1.7 per 10,000 births for microphthalmia [3]. The pathogenesis of congenital anophthalmia is multifactorial, encompassing chromosomal abnormalities, genetic defects, and intrauterine environmental exposures, including teratogenic agents and infections. It may present either as an isolated defect or in association with complex syndromic conditions [2]. Genetic factors play a major role in the pathogenesis of anophthalmia and microphthalmia, with mutations in genes such as SOX2, OTX2, and PAX6 frequently reported. Syndromic forms associated with chromosomal abnormalities, including trisomy 13 and trisomy 18, are also well recognized [3]. Recent advances in prenatal imaging have facilitated the early diagnosis of congenital anophthalmia, while management primarily relies on orbital prosthetic rehabilitation [2]. We report a rare case of isolated bilateral congenital anophthalmia in a newborn with a positive family history and no associated systemic abnormalities, highlighting the clinical and genetic challenges associated with this condition.

## Case presentation

A full-term male neonate, born to consanguineous parents (second-degree consanguinity), was admitted to our neonatal unit on day two of life for assessment and management of bilateral anophthalmia. He was born to a 31-year-old mother, gravida 2 para 2, with no significant medical or surgical history; the couple's first child was healthy and had no congenital or ocular abnormalities. The pregnancy was reported as regularly followed in antenatal care, with delivery occurring by medically assisted vaginal birth and an uneventful perinatal course. However, routine prenatal biological investigations were not performed during pregnancy. A second-trimester morphological ultrasound scan was reported as unremarkable, with no structural abnormalities identified.

Family history revealed several similar cases, with anophthalmia reported in three cousins within the shared parental lineage, raising the possibility of a hereditary component (Figure 1).

**Figure 1:**
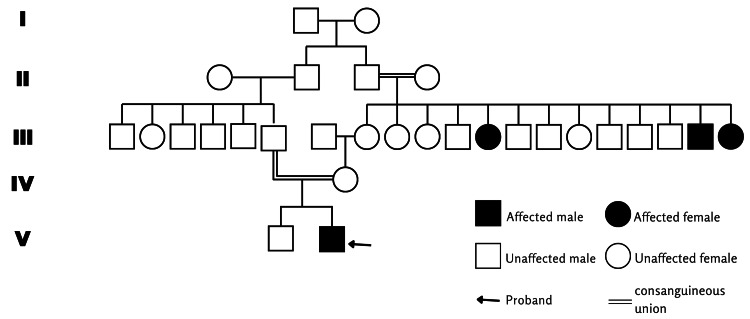
Family pedigree of the newborn showing multiple affected relatives with congenital anophthalmia. The proband is indicated by an arrow. Squares represent males, circles represent females, filled symbols indicate affected individuals, and open symbols indicate unaffected individuals.

Clinical examination revealed bilaterally closed palpebral fissures with absence of ocular globe prominence (Figure 2). No orbital globe structures were palpable on either side. 

**Figure 2:**
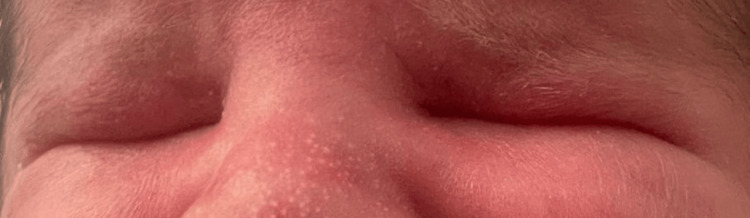
Clinical appearance of bilateral congenital anophthalmia Absent ocular globe prominence and closed palpebral fissures are visible bilaterally.

Ocular ultrasound suggested right microphthalmia with absence of the left globe (Figure 3). Brain MRI, however, showed bilateral anophthalmia associated with hypoplasia of the optic nerves and extraocular muscles, along with corpus callosum hypoplasia (Figure 4).

**Figure 3:**
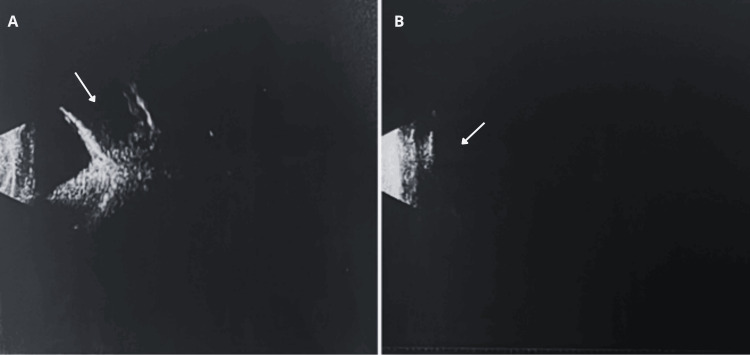
Ocular ultrasound findings. (A) Right orbit showing a rudimentary ocular structure suggestive of microphthalmia (arrow). (B) Left orbit demonstrating absence of the ocular globe, consistent with anophthalmia (arrow).

**Figure 4:**
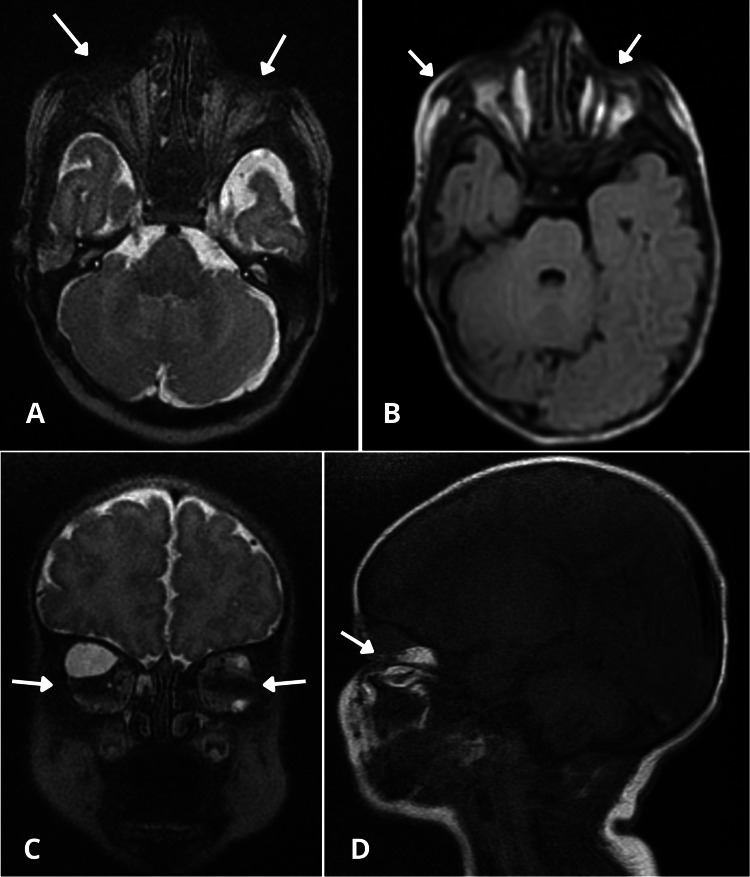
Orbital magnetic resonance imaging demonstrating bilateral clinical anophthalmia. (A, B) Axial T2- and T1-weighted images showing bilateral absence of normally developed globes with severe orbital hypoplasia (arrows). (C) Coronal T2-weighted image revealing bilateral rudimentary ocular remnants within the orbits (arrows). (D) Sagittal image confirming a vestigial ocular structure (arrow).

Peripheral blood karyotype analysis was normal (Figure 5). Aesthetic surgical management with orbital prosthesis placement was proposed after adequate weight gain of the newborn. 

**Figure 5:**
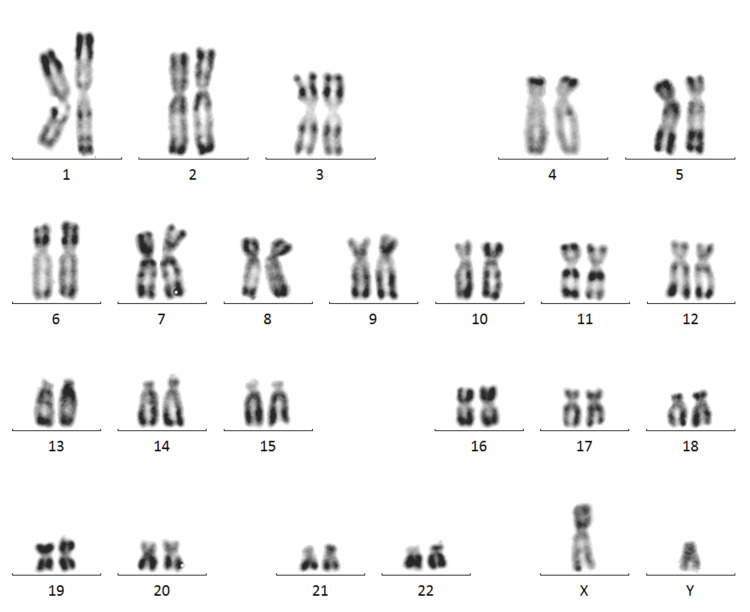
Peripheral blood karyotype showing a normal male karyotype (46,XY).

## Discussion

Congenital anophthalmia refers to the clinical absence of the ocular globe at birth. It is a rare malformation with a variable reported incidence [2]. The combined prevalence of congenital anophthalmia and microphthalmia is estimated at 2.5-3.2 per 10,000 live births [3]. No significant association with sex or ethnic background has been reported [2]. 

The etiology of anophthalmia is multifactorial, involving both genetic and environmental determinants [4]. Anophthalmia and microphthalmia exhibit marked genetic heterogeneity. They may be inherited through autosomal dominant or autosomal recessive patterns, or arise secondary to chromosomal aberrations, most notably trisomy 13 and trisomy 18. In such cases, anophthalmia is often associated with severe brain malformations or midline developmental defects [2]. Most non-syndromic cases occur sporadically [5,6]. 

The main gene implicated in anophthalmia is SOX2, followed by OTX2, RAX, FOXE3, and PAX6 [3,4]. 

Genetic counseling in congenital anophthalmia may be challenging because of the marked genetic heterogeneity, incomplete penetrance, and considerable phenotypic variability associated with these conditions [4]. Identification of a causal genetic anomaly can enable more accurate recurrence risk assessment and provide opportunities for prenatal or preimplantation genetic testing [4]. Although advanced genetic testing could not be performed in the present case because of limited resources, genetic counseling was provided and remains an essential component of care even in the absence of a confirmed molecular diagnosis. 

Several environmental risk factors have been associated with the pathogenesis of anophthalmia and microphthalmia. The most commonly reported include infectious agents (rubella, cytomegalovirus, and toxoplasmosis), exposure during pregnancy to teratogenic substances (alcohol, tobacco, retinoids, thalidomide, ionizing radiation, and excessive solvents), gestational diabetes, and vitamin A deficiency [3,4,7]. No maternal history suggestive of these risk factors was identified. 

Anophthalmia can be diagnosed either prenatally or postnatally based on a combination of clinical examination, imaging studies, and genetic testing. In the present case, no prenatal diagnosis was established. 

The increasing use of advanced antenatal imaging techniques, including fetal magnetic resonance imaging as a second-line modality from 12 weeks of gestation, may provide additional diagnostic value [2,8]. This is exemplified by Dufit et al., who described a case of fetal anophthalmia associated with multiple malformations identified through fetal MRI, resulting in termination of pregnancy [9]. 

Bilateral anophthalmia is less common than unilateral disease [2] but is more frequently associated with syndromic, chromosomal, or monogenic disorders, with a molecular diagnosis identified in up to 70-80% of bilateral anophthalmia or severe microphthalmia cases compared with less than 10% of unilateral cases [5,6]. 

Furthermore, approximately 90% of patients with anophthalmia present with associated malformations [2]. Consequently, in any case of congenital anophthalmia or extreme microphthalmia, a thorough pediatric assessment is required to detect potentially severe associated anomalies [2]. The most commonly reported syndromes include Patau, Fraser, Fryns, Waardenburg, and Matthew-Wood syndromes [1,8,10]. In the present case, comprehensive evaluation, including TORCH screening, abdominopelvic ultrasonography, transfontanellar ultrasonography, echocardiography, and thyroid function testing, revealed no associated systemic abnormalities. The coexistence of bilateral anophthalmia, a positive family history, and the absence of clinically detectable syndromic manifestations makes this case particularly unusual, given the high frequency of associated anomalies reported in the literature.

Even in the absence of clinically visible ocular tissue, histological studies have demonstrated residual neuroectodermal remnants in some cases. Consequently, the terms “true anophthalmia,” “clinical anophthalmia,” and “extreme microphthalmia” actually represent a phenotypic continuum rather than distinct entities [11]. 

Clinically, microphthalmia is generally defined as an ocular axial length that is two standard deviations below the age-adjusted population mean, typically corresponding to less than 21 mm in adults. Simple microphthalmia refers to a small but otherwise structurally normal eye. This term is often used interchangeably with nanophthalmia, although the latter is usually reserved for cases featuring microcornea, an axial length shorter than 18 mm, and high hypermetropia (≥ 8 diopters). The thickened sclera observed in these eyes, along with consequent changes in choroidal blood flow, is thought to account for the increased risk of uveal effusion and choroidal detachment [11].

Microphthalmia may also occur in association with other ocular malformations, in which case it is classified as complex microphthalmia. These associated anomalies can involve the anterior segment (such as sclerocornea or Peters anomaly) and/or the posterior segment (such as persistent hyperplastic primary vitreous or retinal dysplasia) [11].

Both anophthalmia and microphthalmia can present as isolated conditions or as part of a syndrome, with syndromic forms accounting for approximately one-third of cases [11].

The management of anophthalmia is complex and relies on close collaboration among various specialists, including ophthalmologists, oculoplastic surgeons, prosthetists, and psychologists. Parental cooperation remains essential throughout the management process [12]. Treatment decisions should be based on a comprehensive assessment of the underlying cause, the condition of both eyes, the severity of orbital hypoplasia, the patient’s age, and the psychosocial impact of the disease [11].

Early intervention is crucial in children with anophthalmia or microphthalmia without visual potential, to support normal orbitofacial development through artificial stimulation of tissue growth [13]. Orbital rehabilitation should be initiated as soon as possible after birth with the insertion of a conformer, which expands the conjunctival sac and prepares the orbit for prosthetic rehabilitation [14]. When indicated, surgical management may include conjunctival reconstruction, evisceration, orbital expansion implants, or dermis-fat grafting to restore orbital volume and improve cosmetic outcomes [11-13]. 

In developing countries, such complex and sometimes suboptimal management remains difficult to implement due to limited expertise in many centers. In addition, socioeconomic constraints and parental financial limitations restrict access to appropriate care, resulting in significant aesthetic consequences and major psychosocial impact [7]. 

## Conclusions

Congenital anophthalmia and microphthalmia are vision-threatening conditions caused by genetic and/or environmental factors. Prenatal diagnosis is possible in some cases, and early management, with emphasis on aesthetic rehabilitation and psychological support, is essential given the significant visual impairment associated with these conditions. A thorough etiological workup is warranted in all cases of congenital anophthalmia, particularly in the presence of a positive family history. In the present case, the absence of advanced molecular testing limited identification of the underlying genetic cause and precise assessment of recurrence risk, underscoring the need for improved access to genetic investigations in resource-limited settings. Psychological support for affected families remains an essential component of long-term care.
